# Simultaneous transcription of duplicated *var2csa *gene copies in individual *Plasmodium falciparum *parasites

**DOI:** 10.1186/gb-2009-10-10-r117

**Published:** 2009-10-22

**Authors:** Kim JM Brolin, Ulf Ribacke, Sandra Nilsson, Johan Ankarklev, Kirsten Moll, Mats Wahlgren, Qijun Chen

**Affiliations:** 1 Department of Microbiology, Tumor and Cell Biology, Nobels Väg 16, Karolinska Institutet, SE-171 77 Stockholm, Sweden; 2 Swedish Institute for Infectious Disease Control, Nobels Väg 18, SE-171 82, Stockholm, Sweden; 3 Department of Cell and Molecular Biology, Uppsala University, Husargatan 3, SE-751 21 Uppsala, Sweden; 4 Key Laboratory of Zoonosis, Ministry of Education, Jilin University, Xi An Da Lu 5333, Changchun 130062, China; 5 Laboratory of Parasitology, Institute of Pathogen Biology, Chinese Academy of Medical Sciences, Dong Dan San Tiao 9, Beijing 100730, China

## Abstract

**Background:**

Single nucleotide polymorphisms are common in duplicated genes, causing functional preservation, alteration or silencing. The *Plasmodium falciparum *genes *var2csa *and *Pf332 *are duplicated in the haploid genome of the HB3 parasite line. Whereas the molecular function of Pf332 remains to be elucidated, VAR2CSA is known to be the main adhesin in placental parasite sequestration. Sequence variations introduced upon duplication of these genes provide discriminative possibilities to analyze allele-specific transcription with a bearing towards understanding gene dosage impact on parasite biology.

**Results:**

We demonstrate an approach combining real-time PCR allelic discrimination and discriminative RNA-FISH to distinguish between highly similar gene copies in *P. falciparum *parasites. The duplicated *var2csa *variants are simultaneously transcribed, both on a population level and intriguingly also in individual cells, with nuclear co-localization of the active genes and corresponding transcripts. This indicates transcriptional functionality of duplicated genes, challenges the dogma of mutually exclusive *var *gene transcription and suggests mechanisms behind antigenic variation, at least in respect to the duplicated and highly similar *var2csa *genes.

**Conclusions:**

Allelic discrimination assays have traditionally been applied to study zygosity in diploid genomes. The assays presented here are instead successfully applied to the identification and evaluation of transcriptional activity of duplicated genes in the haploid genome of the *P. falciparum *parasite. Allelic discrimination and gene or transcript localization by FISH not only provide insights into transcriptional regulation of genes such as the virulence associated *var *genes, but also suggest that this sensitive and precise approach could be used for further investigation of genome dynamics and gene regulation.

## Abstract 2

Duplicated var2csa genes in one strain of Plasmodium falciparum are simultaneously transcribed, challenging the dogma of mutual exclusive var gene transcription

## Background

Gene duplications, insertions, deletions and single nucleotide polymorphisms (SNPs) are genetic modifications responsible for creation of variable gene families, and contribute to genetic diversity and functional divergence [1]. In humans, gene duplications and deletions have been shown to occur genome wide, thus creating a vast source of genetic variation [2,3]. Genetic alterations are also common throughout the *Plasmodium falciparum *genome, many of which have been shown to correlate with phenotype alterations of this lethal malaria parasite [4-10]. SNPs are commonly introduced in genes upon amplification, causing functional preservation, alteration or dysfunctional alteration/silencing by degenerative mutations of the additional gene copy. To fully understand the impact a particular gene amplification could have on the biology of an organism, it is of major interest to discriminate the paralogs in order to determine the respective gene copy's functionality. The sequence variations introduced upon duplications or evolutionary drift present tools that could enable this discrimination [11].

There is a highly nonrandom distribution of genetic variability in terms of functional classes in *P. falciparum *[5], with the greatest variation in genes coding for proteins associated with the infected red blood cell (iRBC) membrane, which are known to interact with the host immune system [12]. These include the family of *P. falciparum *erythrocyte membrane protein 1 (PfEMP1) proteins, employed by the parasite to sequester in the microvasculature of various organs in the human host. This family, encoded by approximately 60 *var *genes per haploid parasite genome [13-16], presents the parasite with variable surface antigens that enhance the parasite's chances of survival and evasion of the host immune response [17,18]. Earlier studies have indicated that expression of PfEMP1 is mutually exclusive [19,20]; several *var *genes can be transcribed during the ring stage, but in each parasite only one dominant full-length mRNA is transcribed, translated into protein and displayed on the iRBC surface at the mature trophozoite stage [19,21,22].

In pregnancy-associated malaria, parasites bind receptors on the maternal side of the placenta [23], of which chondroitin sulphate A (CSA) is believed to be the main receptor [24,25]. This binding is accomplished using the PfEMP1 VAR2CSA as the main parasite ligand [21]. The gene encoding VAR2CSA (*var2csa*) was recently identified as duplicated in the culture-adapted *P. falciparum *HB3 parasite line [26], originally cloned from the Honduras I/CDC strain in 1983 [27].

The *var2csa *gene is found in nearly all *P. falciparum *isolates [28], and has been suggested to have an ancient origin due to the existence of a *var2csa *ortholog in the genome of the chimpanzee malaria parasite *P. reichenowi*. The gene is unusually conserved compared to other members of the *var *gene family, with observed diversification associated with segmental gene recombination and gene conversion events [26,28]. Sequence polymorphisms between different *var2csa *genes mainly group into segments of limited diversity, with a few basic sequence types within each segment [29]. The two *var2csa *paralogs in the HB3 genome are highly similar in sequence, displaying a nucleotide sequence identity of 89.6% between the complete genes (HB3 genome sequence locus PFHG_05046.1 and PFHG_05047.1 versus locus PFHG_05155.1) and 91.6% between exon 1 (PFHG_05046.1 versus PFHG_05155.1) [30], which encodes the external part of PfEMP1. The sequence differences are found in all parts of the gene, often concentrated into segments, with SNPs of mixed nature (non-synonymous, synonymous and intronic).

Also duplicated in the HB3 genome is the gene *Pf332*, which encodes a suggested surface-associated parasite protein [31]. Pf332 is the largest known malaria protein associated with the iRBC surface but relatively little is known about its molecular function. The protein is suggested to be involved in modulation of RBC rigidity as well as RBC adhesion [32-34] but further studies are needed to scrutinize its function. Parasites with duplicated genes, where functionality is preserved, could potentially be used as tools to elucidate functions of genes of interest. The *var2csa *and *Pf332 *gene copies in HB3 display slight differences in sequence, thereby providing means of discrimination.

Here, we demonstrate a TaqMan real-time PCR assay in which SNPs provide the basis for discrimination between highly similar paralogs in a haploid genome. Using the HB3 parasite line and assays discriminative towards sequence-variable alleles of both *var2csa *and *Pf332*, we show that the different alleles of both genes are readily picked up at the DNA level, with other parasite strains (NF54, FCR3 and Dd2) serving as positive and/or negative controls for the respective alleles. Performing the same analysis on reverse transcribed mRNA, we also show that both paralogs of *var2csa *and *Pf332 *are transcribed by the HB3 parasite, signifying transcriptional functionality of the genes. Furthermore, single cell analyses with both real-time PCR allelic discrimination and RNA-fluorescent *in situ *hybridization (RNA-FISH) with allele-specific probes confirmed that both gene copies of *var2csa *are not only transcribed by individual parasites but also co-localize in the great majority of cells. Highly specific yet facile, the real-time PCR assay provides a useful tool for the investigation of the impact of gene duplications on the biology of *P. falciparum*. Together with localization of genes and corresponding transcripts using FISH, this provides important insights into potential mechanisms regulating surface-expressed antigens on RBCs infected with *P. falciparum*.

## Results and discussion

### Description of designed allelic discriminative *var2csa *and *Pf332 *assays

**Figure 1:**
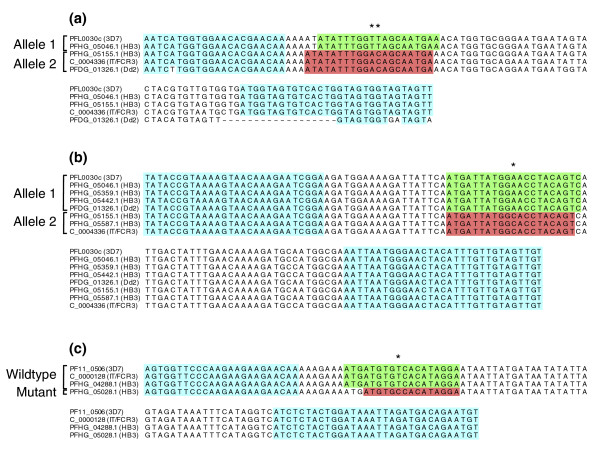
*var2csa *and *Pf332 *alleles in *P. falciparum *genomes and discriminative assay design.  Alignments of *var2csa *and *Pf332 *gene sequences gathered from the fully assembled 3D7 genome and the partially assembled FCR3, Dd2 and HB3 genomes used for allelic discriminative assays. Accession numbers or genome sequence contigs with strain names within parentheses are presented as a means of identification for all alleles. Assays were designed towards two different parts of *var2csa*, **(a) **DBL2x and **(b) **DBL4ε, and **(c) **to exon 1 of *Pf332*. Designed real-time PCR primers are indicated in light blue and probes for allele 1/wild type in green and allele 2/mutant in red. Discriminative SNPs are marked with asterisks.

Two sets of allele-specific real-time primers and TaqMan MGB probes, targeting two different alleles (those encoding the DBL2x and DBL4ε domains, respectively), were designed based on the fully sequenced genomes of *P. falciparum *isolates HB3 and Dd2 [30], and FCR3 and 3D7 [35,36]. 3D7 was originally cloned from NF54 [37] and appears to be isogenic to its ancestor considering similarities in *var *gene sequences, as seen by us in this study as well as by others [21]. The assays were designed to enable detection and discrimination of different *var2csa *variants in FCR3, NF54 and Dd2 and the two *var2csa *paralogs in the HB3 genome (Figure 1a, b). Assay 1 (Figure 1a) was deliberately designed to not amplify *var2csa *of parasite strain Dd2. The allele 2-specific probe of this assay matches the Dd2 *var2csa *sequence perfectly, but due to mismatched annealing sites for both forward and reverse primers, genomic DNA (gDNA) from Dd2 could be used as negative amplification and detection control.

One assay was similarly designed to identify different *Pf332 *variants in NF54, FCR3 and HB3. The discriminative probes were designed towards a non-synonymous SNP altering amino acid 326 in the translated protein from a serine (wild type) to a proline (mutant) - S326P (Figure 1c). This SNP within the recently identified exon 1 of the very large *Pf332 *gene [32] has so far only been identified in the HB3 parasite genome [7,8], preventing the use of any other parasite line as a positive control for the mutant allele. Very little is known about the role of the Pf332 protein and there is no information about the transcription or function of the altered protein. The assay depicted in Figure 1c provides the means for determining the transcription of Pf332.

### Allelic discrimination assay validation

**Figure 2:**
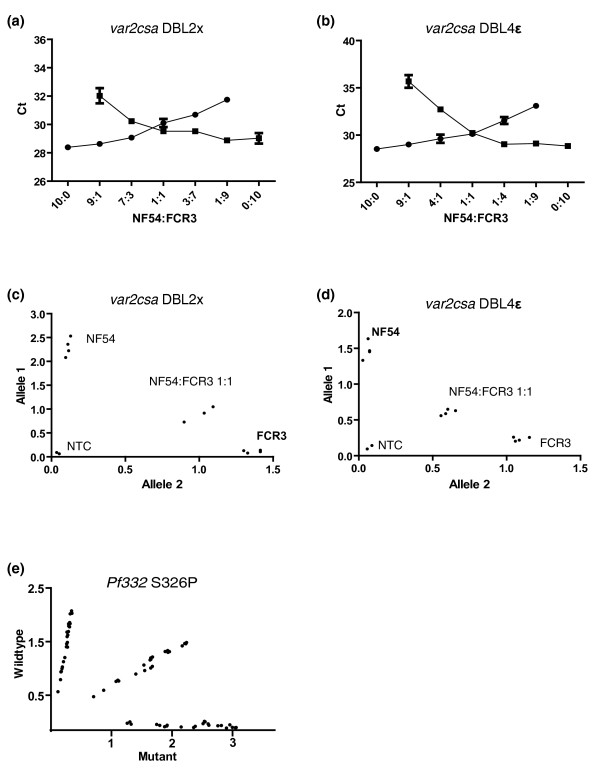
Quality and performance assessment of allelic discrimination assays.  **(a, b) **Shown are cycle thresholds (Ct) achieved using different ratios of NF54 and FCR3 gDNA and discriminative assays for *var2csa *DBL2x (a) and DBL4ε (b). Filled squares represent amplification of allele 1 (detected with FAM) and filled circles indicate amplification of allele 2 (detected with VIC), with error bars representing standard deviations. **(c, d) **Total fluorescence emission, with background deducted, for mixes of NF54 and FCR3 gDNA using the same assays also demonstrates the specificity of *var2csa *probes. **(e) ***Pf332 *allele specificity and concentration dependency is shown using serial dilutions of HB3 gDNA. Amplifications in the presence of only wild-type probe (y-axis), mutant probe (x-axis) or a combination of both (middle) are shown. No template controls (NTC) consistently showed negligible signals in all experiments.

Amplification efficiencies and allele identification specificities were analyzed using gDNA dilutions originating from HB3, NF54, FCR3 and Dd2 parasites. Serial dilutions of HB3 gDNA were employed for amplification efficiency determinations for all three designed assays, yielding highly similar efficiencies within assay components (Additional data file 1). The near identical amplification efficiencies detected therefore provide unbiased amplification of the respective alleles. The specificity and sensitivity of designed primer/probe combinations were analyzed for the *var2csa *alleles using known ratios of NF54 and FCR3 gDNA (known to harbor specific single alleles). The results clearly display ratio-dependent signals using both a cycle threshold (Ct) value approach (detected Ct values for each allele within the assays for each gDNA ratio mixture; Figure 2a, b) as well as a total fluorescence emission approach (amount of detected fluorescence from both probes adjusted for background, that is, post-read compensated with a pre-read signal deduction; Figure 2c, d). Due to the lack of a positive control for one of the *Pf332 *alleles, we used HB3 gDNA for assay validation. For the same serial dilution of gDNA as used for amplification efficiency calculations, three different amplification reactions were performed. The reactions all contained the primers of the assay and either the wild-type detection probe, the mutant-detection probe or a mixture of the two. As shown in Figure 2e, the assay is highly specific for the different alleles, with allele frequencies maintained even at low DNA concentrations.

### Presence of *var2csa *and *Pf332 *alleles in various *P. falciparum *genomes

**Figure 3:**
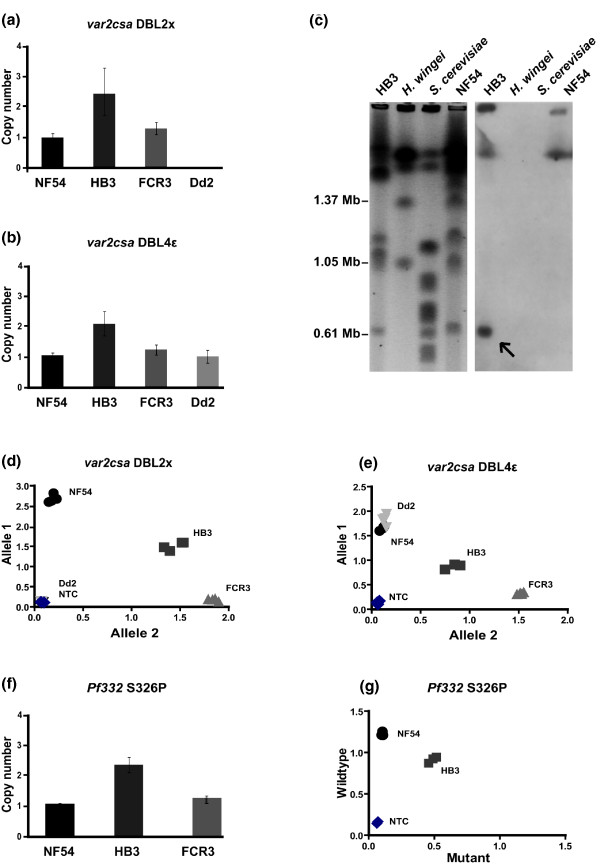
Copy numbers of *var2csa *and *Pf332 *alleles and allelic discrimination.  **(a, b, f) ***var2csa *and *Pf332 *gene copy numbers in various parasite strains are shown relative to the NF54 strain, with error bars representing the confidence interval (CI 95%). Two gene copies were identified in all cases for the HB3 parasite, suggesting that the three additional DBL4ε sequences are due to the partial assembly of this fully sequenced genome. **(c) **PFGE followed by Southern blotting revealed the second *var2csa *copy to be located on chromosome 1 in HB3. An ethidium bromide stained PFGE gel is shown on the left with separated chromosomes from HB3, NF54 and the standards *Hansenula wingei *and *Saccharomyces cerevisiae*; selected chromosome sizes are indicated in megabase-pairs (Mb). The Southern blot shown on the right revealed the var2csa-specific DNA probe to hybridize to chromosome 1 in HB3 (indicated with an arrow). **(d, e) **Discrimination of *var2csa *alleles in gDNA from the indicated parasites showed single allele frequency in NF54, Dd2 (Allele 1) and FCR3 (Allele 2), and double alleles in HB3. **(g) **The same analysis on the S326P mutation in *Pf332 *revealed only the wild-type version in NF54, whereas HB3, with its dual copies, harbors both the wild-type and mutant versions.

In order to confirm the number of gene copies predicted from the fully or partially assembled NF54, FCR3, Dd2 and HB3 genomes, we performed relative copy number analyses of the *var2csa *and *Pf332 *genes. The HB3 genome is supposed to contain two full-length *var2csa *genes and three additional *var2csa *DBL4ε sequences, as shown in Figure 1a, b. Our results indicate that HB3 harbors just the two copies of *var2csa *without the additional DBL4ε sequences (compared to single copies in other parasites; Figure 3a, b), suggesting that the DBL4ε sequences are likely due to the partial assembly of the present HB3 genome. One of the *var2csa *paralogs in HB3 is located on chromosome 12 [26] whereas the location of the second is unknown. In order to determine the chromosomal location of the second *var2csa *copy, we performed pulsed field gel electrophoresis (PFGE) followed by Southern blotting with *var2csa*-specific probes. This revealed the additional *var2csa *paralog in HB3 to be located on chromosome 1 (Figure 3c).

The presence of the sequence-variable alleles was subsequently analyzed using the discriminative method described above. The results show the expected patterns, with single alleles of *var2csa *DBL2x in NF54 and FCR3 and the presence of both allele types in HB3. The negative parasite control (Dd2) showed no amplification of either allele, proving specific *var2csa *amplification and detection (Figure 3d). The assay for the *var2csa *DBL4ε amplification showed identical results, apart from the correct amplification of the expected and correctly primed copy in Dd2 (Figure 3e).

The copy number analysis of *Pf332 *confirmed the duplication of this gene in HB3 (Figure 3f) and these paralogs were similarly proven to be sequence variable (Figure 3g). This gene constitutes one of the largest in the *P. falciparum *genome (18.5 kb) but the number of identified SNPs is relatively low [8,38]. The SNP used here for the discrimination of the two *Pf332 *copies is unique for the HB3 parasite (among the isolates so far sequenced) and could thus be used as a tool for identity determination since cross-contaminated *P. falciparum *strains are relatively common [39]. Taken together, the described *modus operandi *demonstrates the possibility to discriminate duplicated genes based on limited sequence variation.

### The duplicated *var2csa *and *Pf332 *alleles are transcriptionally active

**Figure 4:**
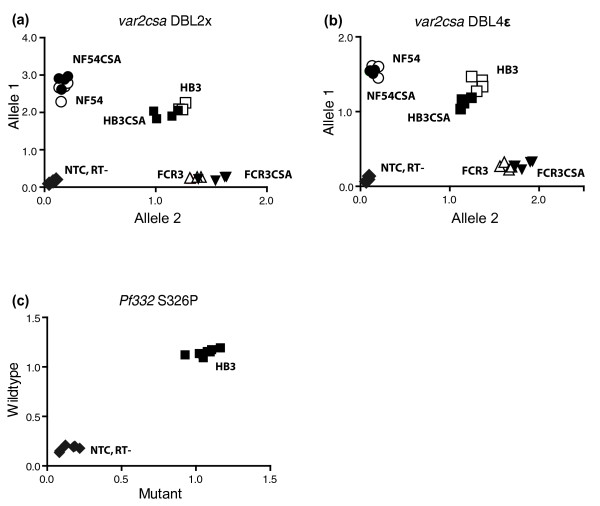
*var2csa *and *Pf332 *allele-specific transcriptional activity. **(a, b) **Transcriptional activity was confirmed for both *var2csa *allele types in HB3 and HB3CSA, and the single allele types of FCR3, FCR3CSA, NF54 and NF54CSA. **(c) **Both *Pf332 *copies in HB3 were also actively transcribed, demonstrating transcriptional functionality in these duplicated genes. Controls with RNA reverse transcribed without addition of reverse transcriptase (RT^-^) and exchange of template for ddH_2_O (NTC) were included in all experiments to prevent signals from gDNA or contaminations from influencing the interpretation of the results.

The presence of alleles at the DNA level says nothing about their transcriptional activity, since silent transcripts are potentially created upon amplification of genes. Hence, *var2csa *transcripts in different parasite lines were analyzed using the same allele-discriminating approach described above. As illustrated in Figure 4a, b, *var2csa *transcripts were detected in all three parasite lines, both before and after CSA selection. Transcriptional activity was confirmed for both *var2csa *genes in HB3 and HB3CSA as well as the respective single alleles in NF54/NF54CSA and FCR3/FCR3CSA (Figure 4a, b). Transcripts of both *Pf332 *copies were similarly present in the HB3 parasite (Figure 4c), signifying preserved functionality of all duplicated alleles, at least at the transcriptional level. These results reflect transcriptional activity in large populations of parasites but give no information about whether both gene copies of *var2csa *and *Pf332 *are expressed in single cells.

### Single mature trophozoites transcribe both *var2csa *gene copies

**Figure 5:**
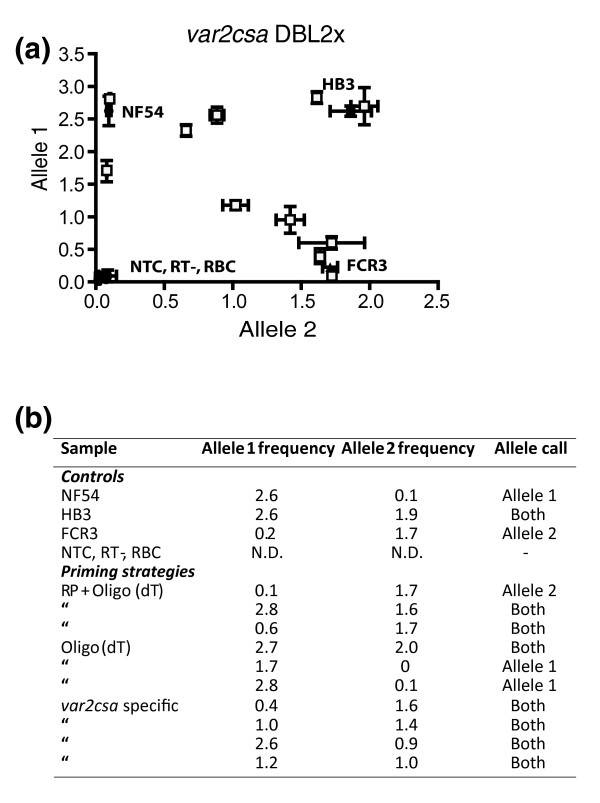
*var2csa *allele transcriptional activity in individual HB3CSA parasites.  Single cell transcription from 11 individual HB3 parasites repeatedly selected for CSA-binding phenotype. Parasites at the mature trophozoite stage (24 ± 4 h p.i.) were subjected to three different priming strategies during the reverse transcription (random primers and oligo(dT), oligo(dT) only and *var2csa*-specific primers). **(a) **Allele frequencies of alleles 1 and 2 for the positive gDNA controls NF54 (filled circles), FCR3 (filled triangles) and HB3 (filled squares) as well as for the 11 cDNA samples (empty square). Negative controls (filled diamonds) with RNA reverse transcribed without addition of reverse transcriptase (RT^-^), exchange of template for ddH_2_O (NTC) and amplifications from uninfected red blood cells (RBCs) were included in all experiments to prevent signals from gDNA, contaminations or unspecific amplifications influencing the interpretation of the results. All data-points represent means of triplicates with standard deviations for each allele expressed as bi-directional error bars. **(b) **Mean allele frequencies and predicted allele calls for all samples and priming strategies described above. N.D., not detected.

Single HB3CSA parasites were collected using micromanipulation and further analyzed with a nested PCR/real-time PCR approach. Surprisingly, both allele types of *var2csa *were observed to be transcribed in individual parasites collected at 24 ± 4 h post-invasion (p.i.; Figure 5a). Transcription of both allele types was readily detected in the majority of cells analyzed, independent of the use of the reverse transcription priming procedure (Figure 5b). For some of the single cells only one of the alleles was detected, either signifying a true difference in transcription pattern among parasites or possibly due to introduced bias in the first PCR amplification. Re-sequencing of the real-time PCR amplified products confirmed the allele calls achieved with the allelic discriminative approach, and revealed *var2csa *sequences exclusively, thus further proving the accuracy and specificity of the assay (data not shown).

**Figure 6:**
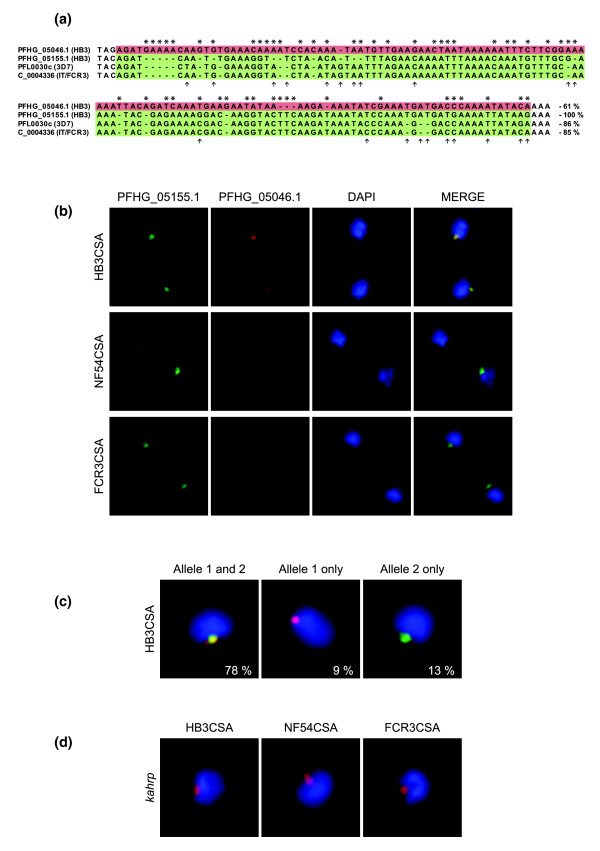
Detection of transcripts from both *var2csa *paralogs in *P. falciparum *parasites as seen by RNA-FISH. **(a) **Alignment of variable *var2csa *sequences serving as template for RNA probes (PFHG_05046.1 and PFHG05155.1) designed to discriminate between the two *var2csa *alleles in HB3CSA (asterisks indicate variable nucleotides between the two alleles in HB3CSA). The sequences of FCR3CSA and NF54CSA display high homology to PFHG_05155.1 in this particular region of the *var2csa *gene, with limited variability (denoted by arrows) allowing detection of the single copies in these parasites at the selected FISH stringency. Percent identical nucleotides between the sequences are displayed (with PFHG_05155.1 as 100%) to the bottom right. **(b) **Representative pictures of the hybridization patterns achieved with the two probes targeting *var2csa *mRNA in indicated parasites. The probe generated from PFHG_05155.1 is displayed in green; the probe towards PFHG_05046.1 in red and parasite nuclei stained with DAPI in blue. Probes towards antisense transcripts consistently showed no hybridization (data not shown). **(c) **Pictures representing the three scenarios observed for detection of *var2csa *allele transcripts in HB3CSA, with frequencies for each scenario (transcripts detected from both paralogs, transcripts detected from only allele 1, and transcripts detected from only allele 2) given as a percentage in each representative picture (n = 92). The great majority of simultaneously transcribed duplicated *var2csa *genes in single HB3CSA cells were exclusively accompanied by the observation of co-localization of the two transcripts in the nuclei. **(d) **Representative hybridization patterns achieved with the control probe targeting the *kahrp *gene (red) in nuclei (blue) of all the CSA-selected parasite lines used. For the *var2csa *hybridizations, the negative control probes towards antisense transcripts of *kahrp *revealed no detection of hybridization.

RNA-FISH was used to visualize and confirm the results from the single cell real-time PCR assays. RNA probes were designed towards one of the most sequence-variable regions of the two *var2csa *paralogs (towards the 5' end) in order to discriminate between them (Figure 6a). The area chosen also presented the possibility of using NF54CSA and FCR3CSA as controls for one of the sequence types in this particular region of *var2csa*. Most HB3CSA parasites (16 ± 4 h p.i.) were indeed shown to have a high abundance of transcripts from both *var2csa *paralogs, whereas NF54CSA and FCR3CSA displayed only transcripts from their single allele types (Figure 6b, c). Control probes towards antisense transcripts of *var2csa *consistently showed no hybridization (data not shown), which is expected due to previous findings of only low levels in CSA-selected FCR3 parasites [40] and a preponderance of *var *gene antisense transcripts appearing at later stages and then mostly limited to the 3' end of exon 1 [41]. In addition, probes generated towards the *kahrp *gene were included as positive controls [42] and hybridized in expected patterns in all three parasites (Figure 6d).

**Figure 7:**
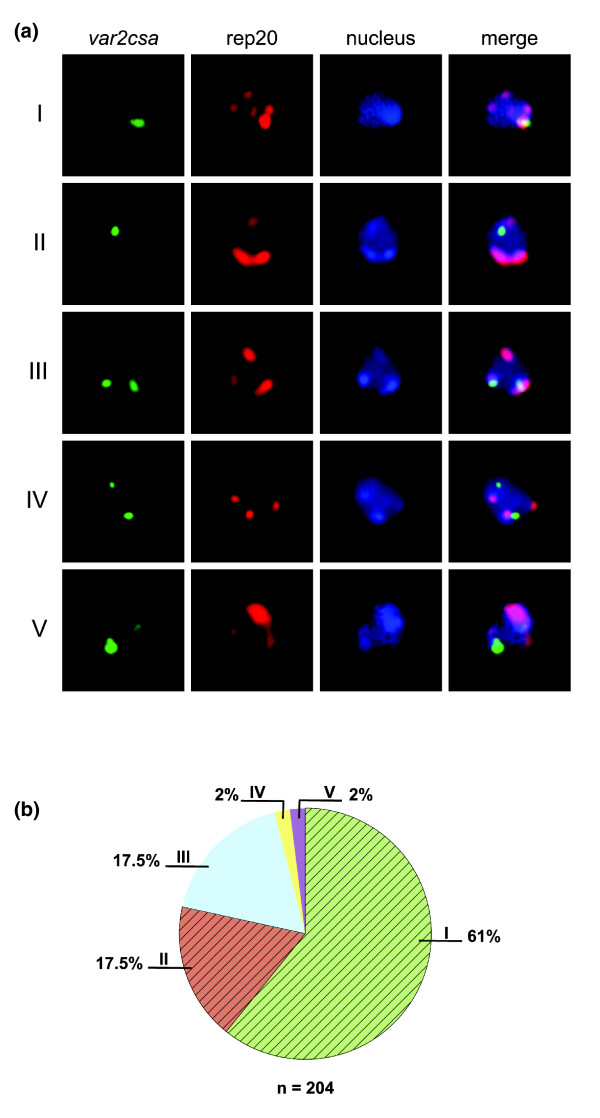
Subnuclear localization of the duplicated *var2csa *genes in HB3CSA parasites.  Representative DNA FISH pictures illustrating the five different localization patterns of both *var2csa *paralogs (green), telomeric clusters (rep20, red) in nuclei stained with DAPI (blue). I, co-localized *var2csa *alleles where both also co-localized with rep20; II, co-localized *var2csa *alleles that did not co-localize with rep20; III, non-co-localized *var2csa *alleles but both co-localized with rep20; IV, non-co-localized *var2csa *alleles with neither allele co-localized with rep20; V, non co-localized *var2csa *alleles with one allele co-localized with rep20. The *var2csa *paralogs were exclusively found towards the rim of the nuclei. **(b) **Quantification of the described localization patterns illustrated in a pie-chart with percentages (n = 204). Co-localized *var2csa *paralogs (I and II) are shaded with stripes and constitute 78.5% of the total.

Apart from confirming simultaneous transcription of the two *var2csa *paralogs in single HB3CSA cells, the RNA-FISH analysis also revealed an intriguing exclusive nuclear co-localization of the two transcripts (Figure 6b, c), this despite the genes being localized on different chromosomes (see above). DNA-FISH, performed in order to further investigate *var2csa *localization in the nucleus of HB3CSA parasites, revealed that the genes also co-localize in the majority of cells (Figure 7, scenarios I and II). The extent of co-localization (78.5%) corresponded very well with the fraction of cells transcribing both paralogs as seen with real-time PCR and RNA-FISH. Previous studies have contradictorily suggested that *var *genes are either distant from and/or adjacent to telomeric ends when active [22,43-46]. Here, the *var2csa *genes in HB3CSA appear both to be distant from and co-localize with Rep20 (representing telomeric clusters), although the latter is more common (Figure 7b).

The transcription of *var *genes at the mature trophozoite stage is presumed to be mutually exclusive [19,20]. However, as shown here, both *var2csa *copies of HB3CSA are simultaneously active in individual cells, challenging the dogma of mutually exclusive transcription of *var *gene family members. Previous studies have indicated that CSA-selected parasites transcribe more than one *var *gene at the mature trophozoite stage [47]. The results of this study are intriguing, especially since both alleles were detected using only oligo(dT) primers in the reverse transcription, suggesting that the transcripts were destined for translation. This was also supported by the common observation of the cytoplasmic localization of both *var2csa *transcripts in cells transcribing both paralogs using RNA-FISH. This is markedly different from the case of *var1csa *(*var*_COMMON_), which is suggested to be simultaneously transcribed along with other *var *genes at the mature trophozoite stage, but to only produce sterile transcripts [48]. The interesting observation of co-localized native *var2csa *genes and transcripts argues for a previously suggested active site of *var *gene transcription [43,45,46]. Whether genes reposition from telomeric clusters within the heterochromatin region into the euchromatin portion of the nuclear periphery upon activation has been both supported [22,43,49] and debated against [44,50]. In the HB3CSA parasites studied here, repositioning (as viewed with Rep20) did not seem necessary for transcriptional activity of the duplicated *var2csa *genes, something that has been shown previously for *var2csa *[22,49]. Despite this controversy, all studies conducted on nuclear localization are consistent with the existence of a specific *var *gene expression site that is apparently able to accommodate more than one active *var *gene, as shown here as well as by Dzikowski *et al*. [45,46], and is perhaps determined by activating and repressive histone methyl modifications [51] rather than by gene position in relation to telomeric clusters.

Even though it challenges the dogma of mutually exclusive *var *gene transcription, simultaneously active duplicated *var2csa *genes could be a special case, since the sequence similarity among different *var2csa *variants is high compared to that of other *var *genes. The fact that one *var2csa *allele in HB3 is located in the subtelomeric region of chromosome 12 [26] whereas the second allele is located on chromosome 1 (Figure 3c) argues that the transcription of *var2csa *is regulated by *trans*-acting factors rather than *cis*-acting elements. This could suggest the presence of *var2csa*-specific transcription factors with preserved DNA-binding regions in the duplicated gene copies. The upstream regions of the *var2csa *paralogs are indeed highly similar (data not shown, but available at Broad Institute of Harvard and MIT [30]), so both are likely bound by analogous DNA binding trans-acting elements, thereby enabling their co-transcription, a so far poorly evaluated mechanism of antigenic variation in *P. falciparum*. However, even though transcriptional activity of both genes is shown here, with transcripts suggested to undergo translation, the function(s) of the sequence polymorphic proteins is not known. Whether both transcripts are indeed translated [7] and proteins exported to the surface of the parasitized erythrocyte and whether functionality is preserved or altered remain to be elucidated in order to understand the impact of this gene duplication on the development of pregnancy-associated malaria.

## Conclusions

Using allele discriminating real-time PCR assays in conjunction with RNA-FISH, with SNPs providing the basis for distinction, we have identified duplicated but slightly sequence variable gene copies in haploid genomes of *P. falciparum*. The different alleles were also proven to be transcriptionally active, an important finding with regard to determining the functionality of duplicated genes. This is the first report in malaria research where allele-specific probes have been used not only to distinguish gene variants and sequence variable gene copies at the genomic level, but also to accurately discriminate allele-specific transcripts. The possibility to differentiate transcripts of the *var2csa *paralogs with the two different methodologies not only showed transcription of two *var *gene copies in single mature trophozoite stage parasites, but also that these co-localized in a great majority of cell nuclei. Not only do these findings challenge the dogma of mutually exclusive *var *gene transcription, they also add complexity to the understanding of the molecular basis of antigenic variation and virulence in pregnancy-associated malaria. The approach can be extended to study other issues related to genetic polymorphisms in malaria - for example, tp determine whether the transcription of members of gene families other than the *var *family is mutually exclusive. Highly specific yet facile and time efficient, this allelic discrimination assay provides a useful tool for the investigation of the impact of gene duplications on the biology of *P. falciparum *as well as mechanisms regulating surface-expressed antigens on red blood cells infected with *P. falciparum*. A more thorough insight into the field of genetic differences and the mechanisms behind these could generate a better understanding of the biology of *P. falciparum*, as well as of the molecular aspects of the pathogenesis of malaria.

## Materials and methods

### Parasite cultivation and CSA-selection

Parasites were maintained in continuous culture according to standard procedures [52]. Parasite lines selected for CSA binding phenotype were repeatedly panned on CSA-coated plastic; 10 μg/ml CSA in phosphate-buffered saline (PBS) was coated on non-tissue culture treated six-well plates overnight in a humid chamber at 4°C. 2% bovine serum albumin in PBS was added for 30 minutes in order to block non-specific binding. Mid-late stage trophozoites were purified using a MACS magnetic cell sorter (Miltenyi BioTec, Bergisch Gladbach, Germany). Purified iRBCs (80 to 90% parasitemia) were washed in RPMI-1640, resuspended in malaria culture medium with 10% human serum and added to CSA-coated wells (approximately 10^8 ^iRBCs per well). Plates were incubated according to standard parasite cultivating procedures for 1 h at 37°C with gentle rocking every 15 minutes. Wells were then washed with malaria culture medium until background binding was low. Finally, 2 ml malaria culture medium with 10% human serum and 100 μl fresh blood was added to each well and plates incubated at 37°C. Parasite suspensions were moved to culture flasks after 24 h and used in downstream experiments. CSA-binding assays were performed according to standard procedures [24].

### Nucleic acid extraction

gDNA was prepared using Easy-DNA Kit (Invitrogen, California, USA) following the supplier's recommendations with minor modifications. The gDNA-containing aqueous phase, once extracted with 25:24:1 phenol:chloroform:isoamyl alcohol (Sigma, St. Louis, Missouri, USA) was RNase treated before one additional round of extraction. Total RNA was harvested at 16 h p.i. for *var2csa *assays and 24 h p.i for *Pf332 *assays using an RNeasy Mini Kit (Qiagen, California, USA). Samples were DNase treated in order to remove contaminating gDNA (Turbo DNase, Ambion, Texas, USA) and reverse transcribed (Superscript III RNase H reverse transcriptase, Invitrogen). For each cDNA synthesis reaction, a control reaction without reverse transcriptase (no template control (NTC)) was performed with identical amounts of template.

### Pulsed field gel electrophoresis and Southern blotting

PFGE using the CHEF Mapper system and pulse field certified agarose (Bio-Rad, California, USA) was performed in order to fractionate HB3 and 3D7 chromosomes. Chromosomes 1 to 4 were separated on a 1% gel in 0.5× TBE buffer with switch times ramped from 60 to 120 s at 6 V cm^-1 ^in a 120° pulse angle for 28 h at 14°C. Chromosomes 5 to 10 were separated on a 1% gel in 0.5× TBE buffer at 14°C with a switch time of 120 s at 4.5 V cm^-1 ^in 120° pulse angle for 22 h followed by a 240 s switch time for 33 h. Chromosomes 11 to 14 were separated on a 0.4% gel in 0.5× TBE buffer with switch times ramped from 120 to 720 s at 2.5 V cm^-1 ^in a 120° pulse angle for 66 h at 18°C. DNA was then transferred to Hybond N+ nylon membranes (GE Healthcare, Stockholm, Sweden) using standard procedures. Southern blotting was performed using an approximately 1-kb double-stranded DNA probe targeting both *var2csa *alleles of HB3, using the primer pair *var2csa *lr1 described in Table 1. In brief, target sequences were amplified in a standard PCR reaction using HB3 gDNA as template followed by a second PCR reaction using the first PCR product as template. The final products were separated on an agarose gel, excised and gel extracted using the QIAquick Gel Extraction Kit (Qiagen), labeled with DIG using DIG High Prime Kit (Roche Applied Science, Indianapolis, USA) and purified using a QIAquick PCR Purification Kit (Qiagen), following the manufacturer's recommendations. Nylon membranes were incubated with 25 ng/ml DIG-labeled probe at 42°C with gentle agitation overnight. The membranes were subsequently washed under high stringency conditions under agitation (2× SSC, 0.1% SDS for 2 × 5 minutes at room temperature followed by 0.5× SSC, 0.1% SDS for 2 × 15 minutes at 68°C) before detection using CSPD (Roche Applied Science) following the recommendations of the manufacturer.

**Table 1:** Primer and probe sequences

Target gene (application)	Primers	Probes
*seryl-tRNA synthesase *(real-time PCR)	F: TATCATCTCAACAGGTATCTACATCTCCTA	NA
	R: TTTGAGAGTTACATGTGGTATCATCTTTT	
*β-tubulin *(real-time PCR)	F: CGTGCTGGCCCCTTTG	NA
	R: TCCTGCACCTGTTTGACCAA	
*var2csa *DBL2x (real-time PCR)	F: AATCATGGTGGAACACGAACAA	6-FAM-ATATTTGGTTAGCAATGAA-MGB (allele 1)
	R: AACTACTACCACTACCAGTGACACTACCAT	6-VIC-ATATATTTGGACAGCAATGA-MGB (allele 2)
*var2csa *DBL4ε (real-time PCR)	F: TATACCGTAAAAGTAACAAAGAATCGGAA	6-FAM-ATGATTATGGAACCTACAGTC-MGB (allele 1)
	R: ACAACTACAACAAATGTAGTTCCCATTAATT	6-VIC-ATGATTATGGCACCTACAGT-MGB (allele 2)
*Pf332 S326P *(real-time PCR)	F: AGTGGTTCCCAAGAAGAAGAACAA	6-FAM-ATGATGTGTCACATAGGA-MGB (wild type)
	R: ACATTCTGTCATCTAATTTATCCAGTAGAGAT	6-VIC-ATGTGCCACATAGGA-MGB (Mutant)
*var2csa *nest 1 (single cell PCR)	F: ACTTGAAAATGTGTGCAAAGGAGTA	NA
	R: TTACCCAGTGGAGACGGAACAT	
*var2csa *lr1 (DNA-FISH/Southern blot)	F: AAAATTACTGTGAATCATTCAGAT	NA
	R: AAGGTGTTTCAGACGAAGTATTAGCAT	
*var2csa *lr2 (DNA-FISH)	F: ACTGTGAATGTTACGAATTGTGGATAA	NA
	R: ACATTTCCCCCTCAATTCCTTT	
*var2csa *PFHG_05046.1_Sp6_a (RNA-FISH, detecting mRNA)	F: TCATTTAGGTGACACTATAGAAGTGTATATTTT GGGTCATCATTTCGATATTT	NA
	R: GAGATGAAAACAAGTGTGAAACAAAATC	
*var2csa *PFHG_05046.1_Sp6_s (RNA-FISH, detecting antisense)	F: TCATTTAGGTGACACTATAGAAGAGATGAAAA CAAGTGTGAAACAAAATCC	NA
	R: TGTATATTTTGGGTCATCATTTCGAT	
*var2csa *PFHG_05155.1_Sp6_a (RNA-FISH, detecting mRNA)	F: TCATTTAGGTGACACTATAGAAGTGCTTATAA TTTTCATCATCATTTGGATATTTATC	NA
	R: CAGATCAATTGAAAGGTTCTAACATTTT	
*var2csa *PFHG_05155.1_Sp6_s (RNA-FISH, detecting antisense)	F: TCATTTAGGTGACACTATAGAAGAGATCAATT GAAAGGTTCTAACATTTTAGAAC	NA
	R: CTTATAATTTTCATCATCATTTGGATATTTATC	
*kahrp*_Sp6_a (RNA-FISH, detecting mRNA)	F: TCATTTAGGTGACACTATAGAAGAGGTTGGTG AACCTGTGGTGCTT	NA
	R: AACTTTAGCACAAAAGCAACATGAA	
*kahrp*_Sp6_s (RNA-FISH, detecting antisense)	F: TCATTTAGGTGACACTATAGAAGAGAACTTTA GCACAAAAGCAACATGAA	NA
	R: GTTGGTGAACCTGTGGTGCTT	

F, forward; NA, not applicable; R, reverse.

### Design of real-time PCR allelic discrimination primers and probes

The fully sequenced genomes of FCR3, Dd2 and HB3 and the partly assembled genome of 3D7 were used as templates for the allelic discrimination assays. Seed sequences from the 3D7 *var2csa *(PFL0030c) and *Pf332 *(PF11_0506) genes [36] were blasted (WashU BLASTN on the BLOSUM62 matrix without low complexity filter) towards the HB3 and Dd2 genomes [30] and the FCR3 genome [35]. Retrieved sequences were analyzed for areas containing moderate sequence variability suitable for conserved primer annealing sites and sequence variable and discriminative probe annealing sites. Primers and probes (MGB probes labeled with either FAM or VIC) for suitable genome contigs were manually designed using Primer Express 3.0 (Applied Biosystems, California, USA). Great care was taken to ensure a high theoretical discrimination possibility, a low risk of primer dimerization and secondary structure formation of all primers and probes (assessed using ΔG estimations in NetPrimer, Premier Biosoft, Palo Alto, California, USA). Specificities of designed assays were confirmed through blasting towards all genomes used as templates.

### Relative gene copy number determination

Relative copy number determinations were performed in quadruplicate in MicroAmp 96-well plates (Applied Biosystems, California, USA) in 20 μl, containing Power SYBR green master mix and primers towards *var2csa *(300 nM of forward and reverse primers of primer pair 1 and 600 nM of forward and reverse primers of primer pair 2), *Pf332 *(300 nM of both forward and reverse primers) and 2 ng of template. As endogenous controls (assumed to exist as single copy genes in every genome) we employed primers for *β-tubulin *(300 nM of forward and reverse primers) and *seryl-tRNA synthetase *(900 nM of forward and reverse primers). Concentrations of primers for the target and reference genes were optimized using serially diluted NF54 gDNA and amplification efficiencies of primers using stated concentrations were sufficiently close to obviate the need for a correction factor. Amplification reactions were carried out in an ABI 7500 real-time PCR system in 40 cycles (95°C for 15 s, and 60°C for 1 minute). Data were analyzed using the Applied Biosystems 7500 system software version 1.3.1 and relative copy numbers were computed according to the ΔΔCt method using a statistical confidence interval of 95%.

### Allelic discrimination

Performance of allelic discrimination assays was also evaluated prior to conducting allele discrimination experiments. Amplification efficiencies of all assays and the allele discrimination capability the *Pf332 *assay were determined using serial dilutions of HB3 gDNA. PCR reactions were performed in quadruplicate in MicroAmp 96-well plates in 20 μl, containing TaqMan buffer with UNG (Applied Biosystems), 900 nm of each forward and reverse primer and 200 nM of either probe. Amplifications were conducted in an ABI 7500 real-time PCR system, starting with a pre-read (for background fluorescence measurement) followed by 40 cycles of amplification (95°C for 15 s, and 60°C for 1 minute) and a final post-read (for total fluorescence emission measurement after amplification). Standard curves for efficiency determination were plotted after the detection threshold was set above the mean baseline value for the first 3 to 15 cycles. Efficiencies of amplification and detection of respective alleles within all assays were shown to be close to identical, proving unbiased allele recognition. Obtained post-read data (adjusted for the background detected in the pre-read) from amplifications of *Pf332 *alleles using the wild-type-detecting FAM-labeled probe, the mutant-detecting VIC-labeled probe and a combination of both probes were used to assess proper allele frequency detection. Proper allele frequency detection was similarly determined for the two *var2csa *allelic discrimination assays using gDNA from NF54 and FCR3 in various mixes. Allele detection frequencies were analyzed using both cycle thresholds for the amplification (with standard deviations expressed as error bars) as well as post-read data. Allelic discrimination experiments were subsequently performed using validated assays on gDNA from NF54, FCR3, Dd2 and HB3 and cDNA from NF54, FCR3 and HB3. Approximately 2 ng of template, the above described primer and probe concentrations, TaqMan buffer with UNG and the same amplification scheme as described above were used. All experiments included NTCs, with ddH_2_O used instead of gDNA for the analysis of the presence of alleles in genomes and reverse transcription control reactions lacking reverse transcriptase for the analysis of allele-specific transcription.

### Single cell nested allelic discrimination

Single iRBCs were picked according to the previously described methodology [19]. In brief, micromanipulation was conducted on highly synchronous HB3CSA cultures (24 ± 4 h p.i.) using a micromanipulator MN-188 (Narishige, Tokyo, Japan), sterile micropipettes (approximately 3 μm internal diameter) and an inverted microscope (Nikon Diaphot 300). A total of 11 iRBCs and 4 RBCs (picked as control) were collected in this manner. Picked cells were deposited in 9 μl drops of 1× Superscript III buffer (Invitrogen) containing 1:30 RNase inhibitor (SUPERase-In, Invitrogen) on multiwell slides pre-treated with dichlorodimethylsilane, and transferred to PCR tubes. Cells were immediately frozen on dry ice before being heated to 94°C for 3 minutes and subsequently DNase treated at 37°C for 30 minutes (rDNase 1, Applied Biosystems, California, USA). Reverse transcription (Superscript III RNase H reverse transcriptase, Invitrogen), was performed according to the manufacturer's recommendations at 50°C for 2 h using three different priming approaches: random primers and oligo(dT)_12-18 _(Invitrogen), oligo(dT)_12-18 _only, or *var2csa*-specific primers (Table 1). For each cDNA synthesis reaction, a control reaction without reverse transcriptase (RT^-^) was performed. We applied a nested PCR approach, initially amplifying an approximately 1-kb fragment of *var2csa *DBL2x with primers (Table 1, *var2csa *nest 1) external to the allele discriminative DBL2x assay. PCR reactions were performed using Platinum Taq DNA Polymerase High Fidelity (Invitrogen) with initial denaturation at 95°C for 5 minutes followed by 35 cycles of amplification (95°C for 30 s, 58°C for 45 s, 68°C for 1 minute 20 s) and final extension at 72°C for 7 minutes. Thereafter, these amplicons were used as templates in real-time PCR allelic discrimination reactions, using primers and probes as described above.

### Fluorescent *in situ *hybridization

RNA-FISH was performed in order to visualize simultaneous transcription of both *var2csa *paralogs in single cells of HB3CSA. Single-stranded antisense and sense RNA probes (Figure 6a) were generated towards one of the most variable regions of the two paralogs using *var2csa *allele-specific primers with Sp6 promoter tails (Table 1) and subsequent *in vitro *transcription. Probes targeting the *kahrp *gene (used as control) were generated using the same procedure. Probes were transcribed in the presence of either fluorescein or biotin and Sp6 RNA polymerase (Roche Applied Science) according to the manufacturer's recommendations. Labeled probes were Dnase treated and purified on Sephadex G-50 fine columns (GE Healthcare, Stockholm, Sweden) and stored at -70°C until use. Highly synchronous HB3CSA, FCR3CSA and NF54CSA parasites (16 ± 4 h p.i.) were isolated from their host erythrocytes using saponin (0.05% w/v) and deposited as monolayers on Denhardt's coated microscope slides. Monolayers were quickly air-dried, immediately fixed in ice-cold 100% ethanol for 2 minutes and subsequently rehydrated through a series of ice cold 90%, 70% and 50% ethanol for 2 minutes each. Slides were further fixed using 4% paraformaldehyde in PBS for 10 minutes at 4°C followed by wash in 2× SSPE (3.0 M Sodium Chloride, 0.2 M Sodium Hydrogen Phosphate, 0.02 M EDTA, pH 7.4) for 3 × 2 minutes. To permeabilize cells, slides were incubated in 0.1 M Tris-HCl with 0.01 M EDTA (pH 8.0) containing 5 μg/ml proteinase K for 10 minutes at room temperature followed by washes in 2× SSPE for 3 × 2 minutes. In order to strip basic proteins from the cells, slides were immersed in 0.2 M HCl for 15 minutes at room temperature followed by washes in 2× SSPE for 3 × 2 minutes. Monolayers were allowed to equilibrate/block in hybridization buffer without probe (10% dextran sulphate, 5× Denhardt's, 50% formamide, 2× SSC, 0.5% SDS, 1:100 RNA protector, 20 μl of 200 mg/ml yeast tRNA in Diethylpyrocarbonate (DEPC) H_2_O). RNA probes were denatured at 65°C for 5 minutes and thereafter immediately put on ice before being resuspended in pre-heated hybridization buffer. Probe mix was then added to slides, which were subsequently covered with a plastic cover and incubated overnight at 42°C. Slides were then washed with 2× SSPE/50% formamide for 15 minutes at 42°C, 2× SSPE for 15 minutes at 42°C, 0.2× SSPE for 15 minutes at 42°C and 0.2× SSPE for 15 minutes at room temperature. Slides were blocked in 1× maelic acid/1× blocking buffer (DIG wash and block buffer set, Roche Applied Science) supplemented with 0.15 M NaCl for 30 minutes at room temperature. Biotin labeled probes were detected with Neutravidin-Texas Red (Molecular Probes, California, USA) diluted 1:500 in 1× maelic acid/1× blocking buffer/0.15 M NaCl for 30 minutes at room temperature. Slides were thereafter washed tice in 1× maelic acid/1× blocking buffer/0.15 M NaCl for 10 minutes at room temperature followed by washing in 1× maelic acid/0.15 M NaCl/0.5% Tween 20 for 2 × 10 minutes at room temperature. Preparations were mounted in Vectashield containing DAPI (Vector Laboratories, California, USA), visualized using a Leica DMRE microscope and imaged with a Hamamatsu C4880 cooled CCD camera.

DNA-FISH on HB3CSA (16 ± 4 h p.i.) was conducted according to the previously described methodology [22], using primers *var2csa *lr1 and lr2 (Table 1) to generate *var2csa *probes recognizing both *var2csa *paralogs in HB3. Synthesis of these probes was performed as described above for Southern blot probes, but labeled with fluorescein using a Fluorescein High Prime Kit (Roche Applied Science) following the instructions of the supplier. Rep20, in a linearized puc9 plasmid, was similarly labeled with biotin using the Biotin High Prime Kit and used for co-localization of the *var2csa *genes with chromosomal telomere ends. Preparations were mounted in Vectashield containing DAPI (Vector Laboratories), visualized using a Leica DMRE microscope and imaged with a Hamamatsu C4880 cooled CCD camera. The physical localization of the two *var2csa *paralogs was determined and quantified for the frequency: co-localized *var2csa *alleles that also co-localized with rep20; co-localized *var2csa *alleles but with no co-localization with rep20; non co-localized *var2csa *alleles where both co-localized with rep20; non-co-localized *var2csa *alleles where neither allele co-localized with rep20; and non-co-localized *var2csa *alleles with only one of the alleles co-localizing with rep20.

## Abbreviations

CSA: chondroitin sulphate A; Ct: cycle threshold; DEPC: Diethylpyrocarbonate; FISH: fluorescent *in situ *hybridization; gDNA: genomic DNA; iRBC: infected red blood cell; NTC: no template control; PBS: phosphate-buffered saline; PfEMP1: *P. falciparum *erythrocyte membrane protein 1; PFGE: pulsed field gel electrophoresis; p.i.: post-invasion; SNP: single nucleotide polymorphism.

## Authors' contributions

UR, KB and QC conceived and designed the experiments. KB, UR, SN and JA performed the experiments. KB, UR, KM and QC analyzed the data. QC and MW contributed reagents/materials/analysis tools. KB, UR and QC wrote the manuscript. All authors read and approved the final manuscript.

## Additional data files

The following additional data are available with the online version of this paper: a figure showing amplification efficiencies of allelic discrimination assays (Additional data file 1).

## Supplementary Material

Additional data file 1 Graphs showing standard curves of amplifications using primer pairs towards **(a) ***var2csa *DBL2X, **(b) ***var2csa *DBL4ε and **(c) ***Pf332 *S326P together with detection by allele-specific FAM- or VIC-labeled probes. Serial dilutions of HB3 gDNA were used in all reactions. Filled squares show amplification detected with FAM-labeled probe and filled circles detection with VIC-labeled probe. Amplification efficiencies for primers and probes within all respective allele assays were sufficiently close to obviate the need for a correction factor. Click here for file
