# Supplementary figures and images for: Simultaneous transcription of duplicated var2csa gene copies in individual Plasmodium falciparum parasites
Source: Genome Biol. 2009 Oct 22;10(10):R117. doi: 10.1186/gb-2009-10-10-r117 (PMC2784332; doi:10.1186/gb-2009-10-10-r117)

Supplementary figure 1

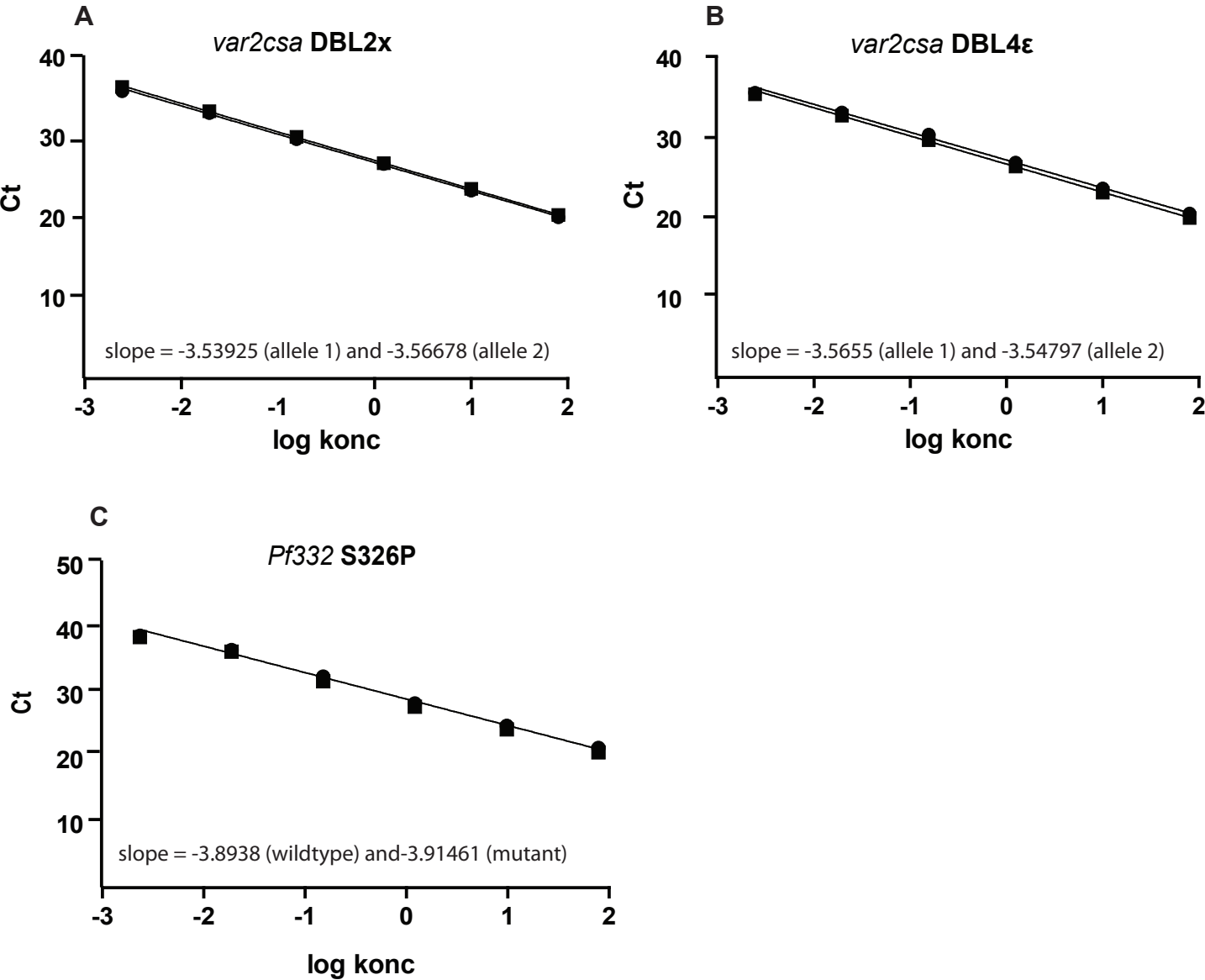

Supplement: Additional data file 1 — Graphs showing standard curves of amplifications using primer pairs towards (a) var2csa DBL2X, (b) var2csa DBL4ε and (c) Pf332 S326P together with detection by allele-specific FAM- or VIC-labeled probes. Serial dilutions of HB3 gDNA were used in all reactions. Filled squares show amplification detected with FAM-labeled probe and filled circles detection with VIC-labeled probe. Amplification efficiencies for primers and probes within all respective allele assays were sufficiently close to obviate the need for a correction factor. [file gb-2009-10-10-r117-S1.pdf]
